# Supplementary material for: Unravelling the mechanism of neurotensin recognition by neurotensin receptor 1
Source: Nat Commun. 2023 Dec 9;14:8155. doi: 10.1038/s41467-023-44010-7 (PMC10710507; doi:10.1038/s41467-023-44010-7)
Supplement: Supplementary file 1 — Supplementary Information [file 41467_2023_44010_MOESM1_ESM.pdf]

Supplementary Information

Unravelling the mechanism of neurotensin recognition by neurotensin receptor 1

Kazem Asadollahi[a,b,c], Sunnia Rajput[b], Lazarus Andrew de Zhang[c,d], Ching-Seng Ang[b], Shuai Nie[b], Nicholas A. Williamson[b], Michael D. W. Griffin[a,b], Ross A. D. Bathgate[a,c], Daniel J. Scott[a,c], Thomas R. Weikl[e], Guy N. L. Jameson[b,f], Paul R. Gooley[a,b]

[a] Department of Biochemistry and Pharmacology, University of Melbourne, Parkville, VIC 3010, Australia

[b] Bio21 Molecular Science and Biotechnology Institute, University of Melbourne, Parkville, VIC 3010, Australia

[c] The Florey, University of Melbourne, Parkville, VIC 3010, Australia

[d] Monash Institute of Pharmaceutical Sciences, Monash University, 381 Royal Parade, Parkville, VIC 3052, Australia

[e] Department of Biomolecular Systems, Max Planck Institute of Colloids and Interfaces, 14476 Potsdam, Germany

[f] School of Chemistry, University of Melbourne, Parkville, VIC 3010, Australia

Supplementary figures

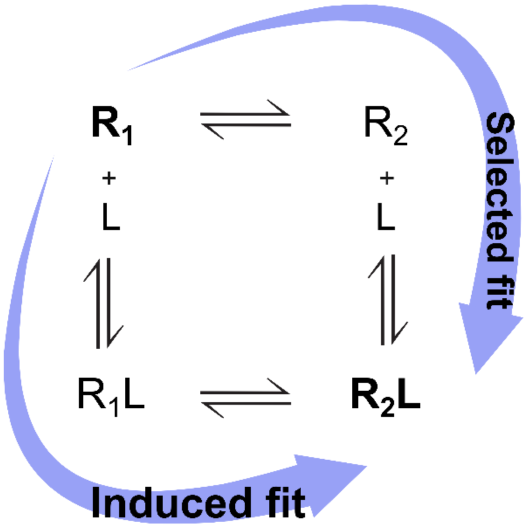

Supplementary Figure 1. Binding mechanisms. Along the induced fit pathway, the conformational change occurs after binding, and along the selected-fit pathway, the conformational change precedes binding. Here R_1 and R_2 represent the ground-state and excited-state conformations of the free protein, L represents the ligand, and R_1L and R_2L represent the excited-state and the ground-state conformations of the bound complex respectively.

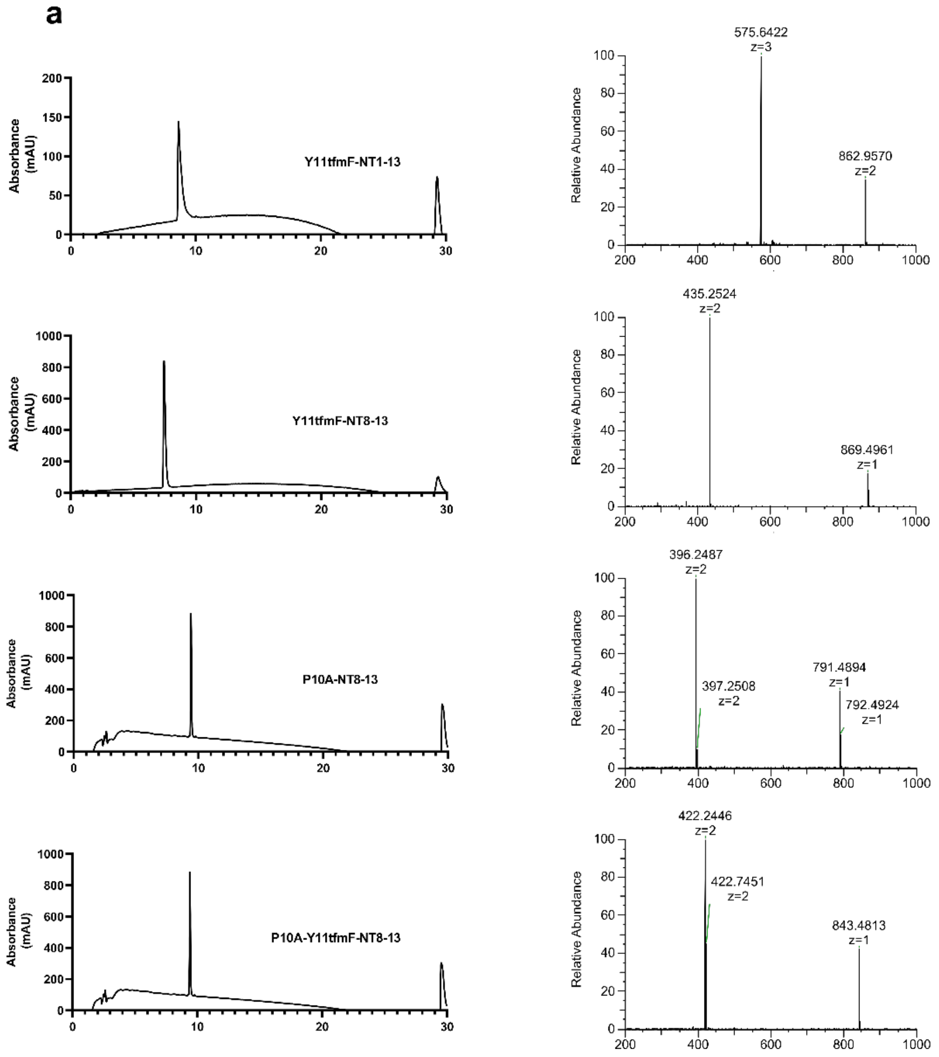

Supplementary Figure 2. The quality control of the synthesized ^{19}F peptide analogues of NT. Peptide purity was assessed by analytical reverse-phase (C18) HPLC (left panels) and the peptide identity by Mass Spectrometry (right panels). The analytical HPLC chromatograms and corresponding MS spectrum are labelled by peptide name.

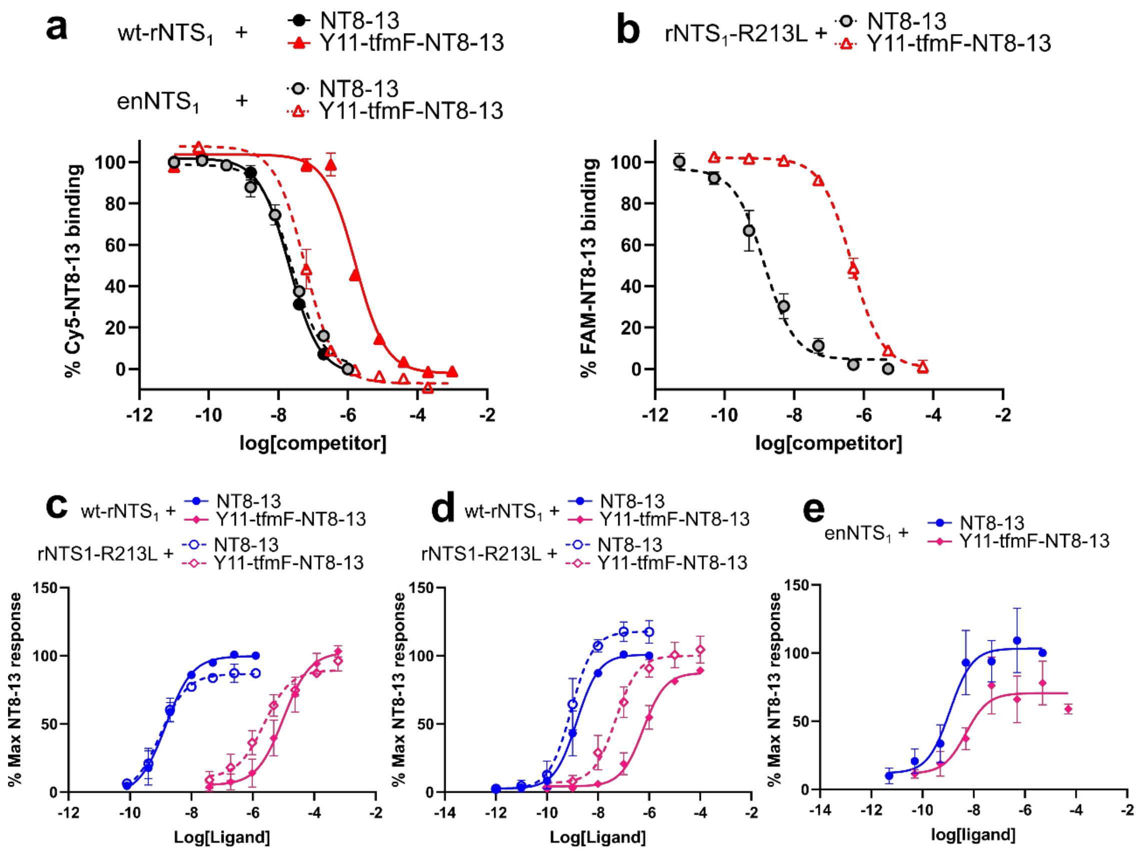

f

	NT8-13			Y11tfmF-NT8-13		
	LogK_i ± SEM (nM)	LogEC50 ± SEM (nM)		LogK_i ± SEM (nM)	LogEC50 ± SEM (nM)	
		β-arrestin	G-protein		β-arrestin	G-protein
wt-rNTS$_1$	-8.289 ± 0.055	-8.814 ± 0.059	-8.834 ± 0.063	-6.300 ± 0.055	-6.179 ± 0.072	-5.005 ± 0.096
rNTS$_1$-R213L	-9.417 ± 0.195	-9.043 ± 0.078	-9.005 ± 0.084	-6.909 ± 0.010	-7.251 ± 0.123	-5.633 ± 0.113
enNTS$_1$	-8.307 ± 0.055	——	-8.896 ± 0.166	-8.082 ± 0.060	——	-8.308 ± 0152

Supplementary Figure 3. Functional characterization of the NT peptides used in this study. The cell-surface binding of peptides to (a) wt-rNTS$_1$ and enNTS$_1$ (b) rNTS$_1$-R213L. The competition binding curves are for NT8-13 (black circles), Y11tfmF-NT8-13 (red triangles). Cy5-labellled NT8-13 and FAM-labelled NT8-13 was used as a fluorescent competitor for investigating the binding. (c) G-protein activation and (d) β-arrestin recruitment assays showing the dose response curves for NT8-13 (blue curves) and Y11tfmF-NT8-13 (pink curves) against wt-rNTS$_1$ (closed symbols/solid line) and rNTS$_1$-R213L (open symbols/dotted line). (e) BRET assays for G-protein activation by enNTS$_1$ in response to NT8-13 (blue) and Y11tfmF-NT8-13 (pink). It is noteworthy that the variant enNTS$_1$ used in this study does not signal through β-arrestin. (f) The equilibrium inhibitory constant (K_i) and half maximal effective concentration (EC50) for the peptides is presented as Log K_i and Log EC50, respectively, ± standard error of mean (SEM). Data points are represented as the mean of quadruplicate experiments (n = 4) ± standard error of mean (SEM) in (a) and triplicate experiments ± SEM for the rest of assays. The signal in the presence of different concentrations of ligand is normalized against the signal of FAM-NT8-13 or Cy5-NT8-13 in the absence of competitor and is presented as percent FAM-NT8-13 or Cy5-NT8-13 binding.

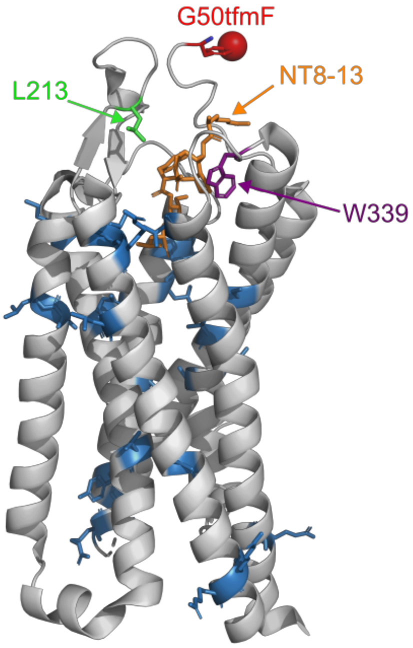

$rNTS_1$	MHLNSSVPQGTPGEPDAQPFSGPQSEMEATFLALSLSNGSGNTSESDTAGPNSDLDVNTD	60
$enNTS_1$	--GSTSESDTAGPNSDLDVNTD	20
$rNTS_1$	IYSKVLVTAIYLALFVVGTVGNSVTAFTLARKKSLQSLQSTVHYHLGSLALSDLLILLLA	120
$enNTS_1$	IYSKVLVTAIYLALFVVGTVGNGVTLFTLARKKSLQSLQSRVDYYLGSLALSSLLILLFA	80
$rNTS_1$	MPVELYNFIWVHHPWAFGDAGCRGYYFLRDACTYATALNVASLSVERYLAICHPFKAKTL	180
$enNTS_1$	LPVDVYNFIWVHHPWAFGDAGCKGYYFLREACTYATALNVVSLSVERYLAICHPFKAKTL	140
$rNTS_1$	MSRSRTKKFISAIWLASALLAIPMLFTMGLQNRSGDGTHPGGLVCTPIVDTATVKVVIQV	240
$enNTS_1$	MSRSRTKKFISAIWLASALLSLPMLFTMGLQNLSGDGTHPGGLVCTPIVDTATLRVVIQL	200
$rNTS_1$	NTFMSFLFPMLVISILNTVIANKLTVMVHQAAEQGRVCTVGTHNGLEHSTFNMTIEPGRV	300
$enNTS_1$	NTFMSFLFPMLVASILNTVIARRLTVMVHQAAEQARVSTVGTHNGLEHSTFNMTIEPGRV	260
$rNTS_1$	QALRHGVLVLRAVVIAFVVCWLPYHVRRLMFCYISDEQWTTFLFDFYHYFYMLTNALFYV	360
$enNTS_1$	QALRRGVLVLRAVVIAFVVCWLPYHVRRLMFVYISDEQWTTALFDFYHYFYLLSNALVYV	320
$rNTS_1$	SSAINPILYNLVSANFRQVFLSTLACLCPGWRHRRKKRPTFSRKPNSMSSNHAFSTSATR	420
$enNTS_1$	SAAINPILYNLVSANFRQVFLSTLASLSPGWRHRRKKRPTFSRKPNSMSSNHAFSTAS--	378
$rNTS_1$	ETLY 424	
$enNTS_1$	---- 378	

Supplementary Figure 4. Alignment of wt-$rNTS_1$ and $enNTS_1$. $enNTS_1$was evolved by directed evolution methods from $rNTS_1$.[1] Thermostabilizing mutations are highlighted in blue on the crystal structure of $rNTS_1$ (PDB: 4xee). Residue 213 which is leucine in $enNTS_1$ and arginine in $rNTS_1$ is highlighted in green, G50 located in the N-terminal region that was mutated to the unnatural amino acid trifluoromethyl-phenylalanine (p-tfmF) is in red. W339 whose fluorescence we speculate is sensitive to NT is in purple. NT is shown in orange.

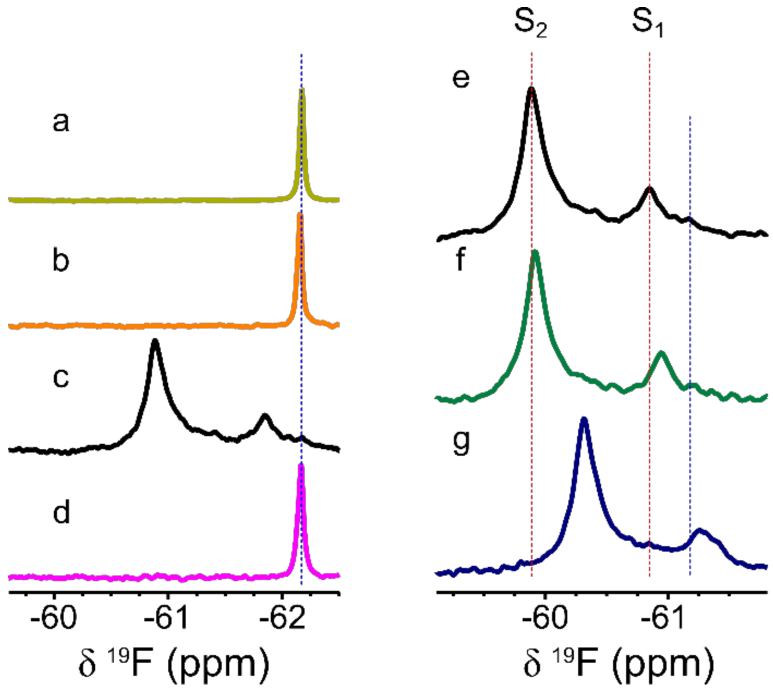

Supplementary Figure 5. ^{19}F-NMR spectra of Y11tfmF-NT analogues in complex with enNTS$_1$. ^{19}F NMR spectra of Y11tfmF-NT1-13 (a) in the free state in solution, (b) in phosphate buffer supplemented with 0.4% DDM, (c) in complex with enNTS$_1$ and (d) in the presence of enNTS$_1$ and excess amounts of unlabelled NT. These spectra show that binding of Y11tfmF-NT to enNTS$_1$ results in two signals. ^{19}F NMR spectra (e) of Y11tfmF-NT1-13, (f) Y11tfmF-NT8-13 and (g) P10A-Y11tfmF-NT8-13 in complex with enNTS$_1$. The major populated states in receptor complexes of Y11tfmF-NT1-13 and Y11tfmF-NT8-13 are assigned as S$_1$ and S$_2$ (red dashed lines). The presence of both peaks in the truncated peptide and P10A mutant proposes that the presence of the two major peaks in the bound states are not due to cis/trans isomerization of prolines. The blue dotted lines in a-g represent the signal from the free peptide. All the spectra are referenced to the signal of 20 μM TFA internal standard at -75.66 ppm.

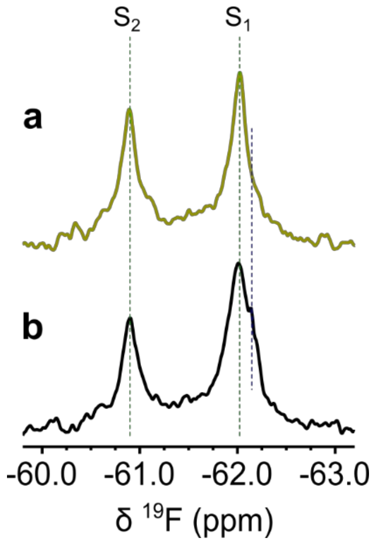

Supplementary Figure 6. Effect of G protein binding on the conformational dynamics of extracellular surface of enNTS$_1$-R213. ^{19}F spectrum of (a) Y11tfmF-NT8-13 in the presence of excess enNTS$_1$-R213 and (b) Y11tfmF-NT8-13 in the presence of excess enNTS$_1$-R213 in the presence of 5-fold excess chimeric Gα_{iq} that was expressed and purified as previously described.[2] The blue dotted line shows the free peptide.

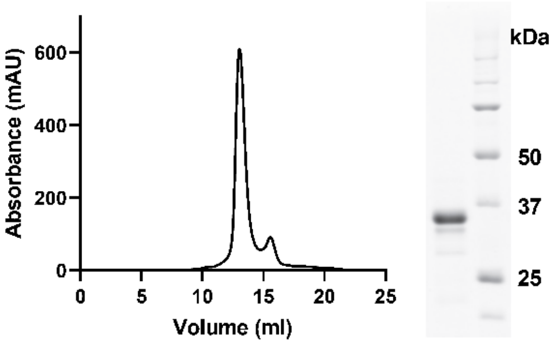

Supplementary Figure 7. The G50tfmF-NTS$_1$ is expressed in *E. coli* and purified to homogeneity. The size exclusion chromatogram of G50tfmF-R213-NTS$_1$ on a Superdex S200 10/30 increase column purified from *E. Coli*. The pooled fractions containing receptor peak were loaded on SDS-PAGE. The marker sizes have been noted next to the marker lane. Source data for this figure are provided as a Source Data file.

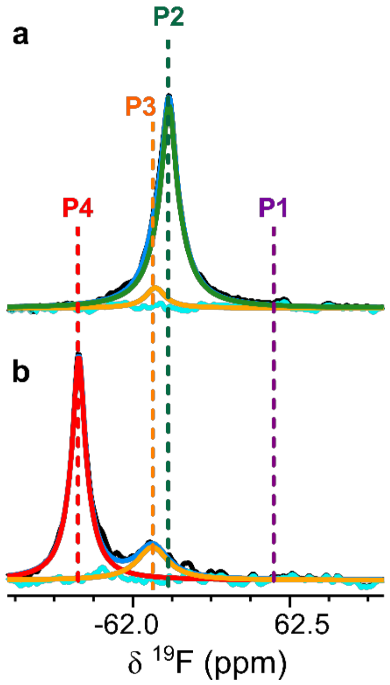

Supplementary Figure 8. ^{19}F NMR spectra of G50tfmF-enNTS$_1$-P51A-R213. (a) The ^{19}F NMR spectra of apo G50tfmF-enNTS$_1$-P51A-R213 and (b) in complex with NT8-13. In comparison to G50tfmF-enNTS$_1$-R213 the signal for P1 is lost, indicating that P1 and P2 are assigned respectively to the cis and trans isomer states of P51. Addition of agonist induces the new population P4 with a small P3 population as observed for G50tfmF-enNTS$_1$-R213. The blue and cyan line show the sum and residuals of the deconvoluted spectra, respectively.

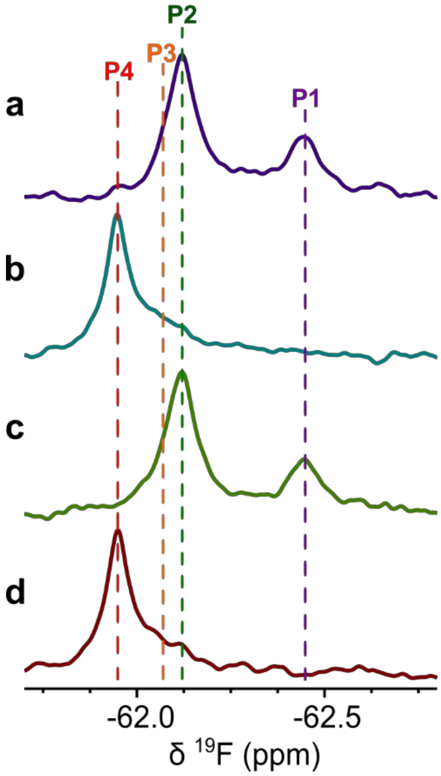

Supplementary Figure 9. Effect of G protein on the ^{19}F NMR spectrum of G50tfmF-enNTS$_1$-R213. ^{19}F spectra of (a) apo G50tfmF-enNTS$_1$-R213, (b) G50tfmF-enNTS$_1$-R213 in the presence of excess NT8-13, (c) G50tfmF-enNTS$_1$-R213 in the presence of 5-fold excess chimeric Gα_{iq} and (d) G50tfmF-enNTS$_1$-R213 in the presence of excess NT8-13 and 5-fold excess chimeric Gα_{iq}.

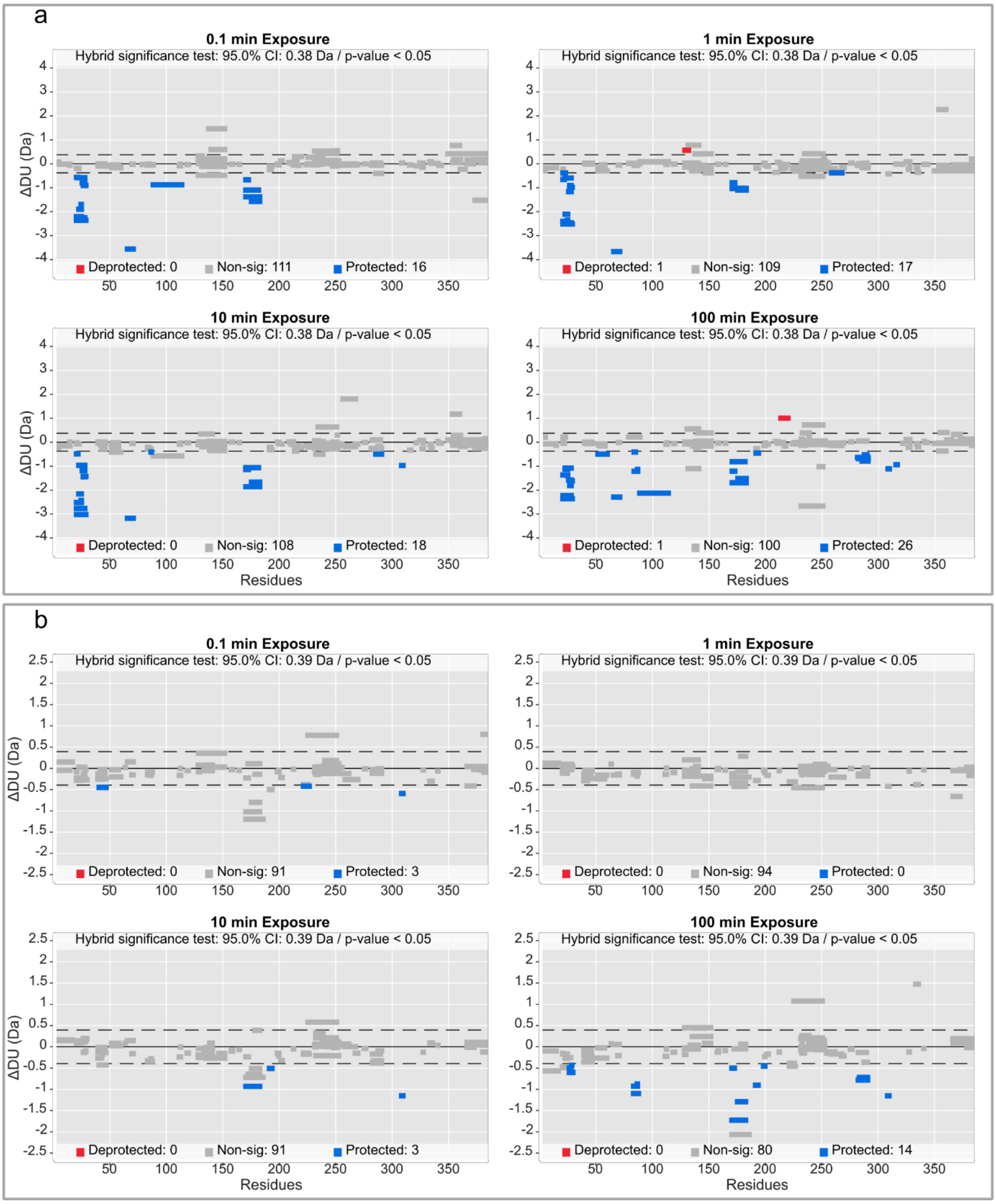

Supplementary Figure 10. Statistical analysis of HDX-MS data. Hybrid Woods differential plots were generated using Deuteros 2.0[3] to identify peptides with significant differences in hydrogen-deuterium uptake (confidence interval of 95%) for each time point in different receptor states. Hybrid Woods Differential plots comparing the deuterium uptake of enNTS$_1$ in (a) the presence and absence of NT8-13 and (b) the presence and absence of SR142948A. Regions highlighted in blue indicate protection upon ligand binding. The dashed lines indicate the confidence limit (Cl).

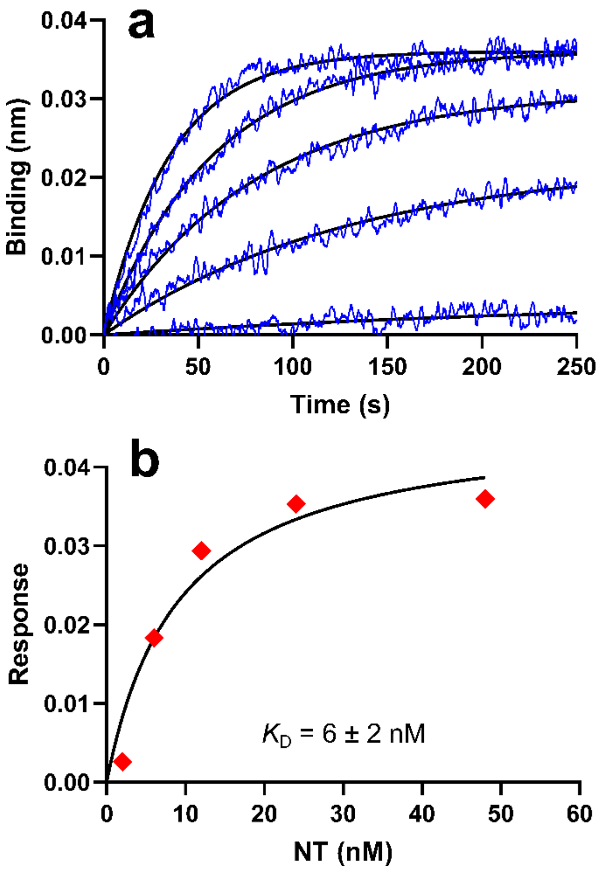

Supplementary Figure 11. Binding of NT to enNTS$_1$. (a) Real-time traces of binding of NT (from 2 to 48 nM) to C-terminal avi-tagged enNTS$_1$ immobilized on the surface of a streptavidin Octet sensor. (b) Saturation binding curve of NT interaction with enNTS$_1$. Source data for this figure are provided as a Source Data file.

Supplementary Table 1. The chemical shift and population of conformational states of N-terminally labelled receptor in the apo and ligand bound states.

		δ ^{19}F (ppm)				Population (%)			
**Receptor**	**Ligand**	**P1**	**P2**	**P3**	**P4**	**P1**	**P2**	**P3**	**P4**
G50tfmF-enNTS$_1$	-	-62.45	-62.12	-62.09	-	31.8	53.8	14.4	-
	NT8-13	-	-	-62.06	-61.9	-	-	10	90
G50tfmF-enNTS$_1$-R213	-	-62.45	-62.12	-62.08	-	31.8	64	4.2	-
	NT8-13		-62.11	-62.06	-61.95		9.3	1.3	89.4
	SR142958A	-62.43	-62.11	-62.06		29.3	49	21.7	
G50tfmF-enNTS$_1$-P51A-R213	Apo	-	-62.11	-62.07	-	-	91	9	-
	NT8-13	-	-	-62.06	-61.83			23	77

References

1. Bumbak, F. et al. Optimization and (13)CH3 methionine labeling of a signaling competent neurotensin receptor 1 variant for NMR studies. *Biochim Biophys Acta Biomembr* **1860**, 1372-1383 (2018).
2. Bumbak, F. et al. Stabilization of pre-existing neurotensin receptor conformational states by beta-arrestin-1 and the biased allosteric modulator ML314. *Nat Commun* **14**, 3328 (2023).
3. Lau, A.M., Claesen, J., Hansen, K. & Politis, A. Deuteros 2.0: peptide-level significance testing of data from hydrogen deuterium exchange mass spectrometry. *Bioinformatics* **37**, 270-272 (2021).
