# Supplementary material for: Unravelling the mechanism of neurotensin recognition by neurotensin receptor 1
Source: Nat Commun. 2023 Dec 9;14:8155. doi: 10.1038/s41467-023-44010-7 (PMC10710507; doi:10.1038/s41467-023-44010-7)
Supplement: Supplementary file 3 — Reporting Summary [file 41467_2023_44010_MOESM3_ESM.pdf]

nature portfolio

Corresponding author(s): Paul R Gooley

Last updated by author(s): Oct 10, 2023

Reporting Summary

Nature Portfolio wishes to improve the reproducibility of the work that we publish. This form provides structure for consistency and transparency in reporting. For further information on Nature Portfolio policies, see our Editorial Policies and the Editorial Policy Checklist.

Statistics

For all statistical analyses, confirm that the following items are present in the figure legend, table legend, main text, or Methods section.

n/a	Confirmed	
☐	☒	The exact sample size (*n*) for each experimental group/condition, given as a discrete number and unit of measurement
☐	☒	A statement on whether measurements were taken from distinct samples or whether the same sample was measured repeatedly
☒	☐	The statistical test(s) used AND whether they are one- or two-sided<br>*Only common tests should be described solely by name; describe more complex techniques in the Methods section.*
☒	☐	A description of all covariates tested
☒	☐	A description of any assumptions or corrections, such as tests of normality and adjustment for multiple comparisons
☐	☒	A full description of the statistical parameters including central tendency (e.g. means) or other basic estimates (e.g. regression coefficient) AND variation (e.g. standard deviation) or associated estimates of uncertainty (e.g. confidence intervals)
☒	☐	For null hypothesis testing, the test statistic (e.g. *F*, *t*, *r*) with confidence intervals, effect sizes, degrees of freedom and *P* value noted<br>*Give P values as exact values whenever suitable.*
☒	☐	For Bayesian analysis, information on the choice of priors and Markov chain Monte Carlo settings
☒	☐	For hierarchical and complex designs, identification of the appropriate level for tests and full reporting of outcomes
☒	☐	Estimates of effect sizes (e.g. Cohen's *d*, Pearson's *r*), indicating how they were calculated

Our web collection on statistics for biologists contains articles on many of the points above.

Software and code

Policy information about availability of computer code

Data collection	Topspin vs3.2.7 (Bruker); ChronosHDX (Trajan); Pro-Data SX version 2.5 (Applied Photophysics); Octet BLI Discovery 12.2.1.18 (Sartorius)
Data analysis	GraphPad Prism 9.3.1 (Graphpad Software, San Diego, CA); Topspin vs3.4 (Bruker); MNova (version: 10.0.2-15465, Mestrelab Research); DynamX 3.0 (Waters); Deuteros 2.0; Pro-Data SX version 2.5 (Applied Photophysics); Mathematica 13; Octet BLI Analysis 12.2.1.3 (Sartorius); Protein Lynx Global Server (PLGS) v3.0;

For manuscripts utilizing custom algorithms or software that are central to the research but not yet described in published literature, software must be made available to editors and reviewers. We strongly encourage code deposition in a community repository (e.g. GitHub). See the Nature Portfolio guidelines for submitting code & software for further information.

Data

Policy information about availability of data

All manuscripts must include a data availability statement. This statement should provide the following information, where applicable:

- Accession codes, unique identifiers, or web links for publicly available datasets
- A description of any restrictions on data availability
- For clinical datasets or third party data, please ensure that the statement adheres to our policy

Our data availability statement: The mass spectrometry proteomics data have been deposited to the ProteomeXchange Consortium via the PRIDE75 partner repository with the dataset identifier PXD045464. Other data available from the corresponding author upon reasonable request. The NMR datasets (Figure 1, 2, S5, S6, S8, S9), the stopped-flow traces analysed in Figure 4b and Cell assay data (Figure S3) are available on Zenodo and can be accessed via https://doi.org/10.5281/

zenodo.10146660. PDB files referenced in this manuscript are available at the Protein Data Bank (https://www.rcsb.org/): 4XEE [https://doi.org/10.2210/pdb4XEE/pdb], 6YVR [https://doi.org/10.2210/pdb6YVR/pdb], 4BUO [https://doi.org/10.2210/pdb4BUO/pdb], 6Z4Q [https://doi.org/10.2210/pdb6Z4Q/pdb], 6Z66 [https://doi.org/10.2210/pdb6Z66/pdb], 6Z4S [https://doi.org/10.2210/pdb6Z4S/pdb], 6Z8N [https://doi.org/10.2210/pdb6Z8N/pdb], 6ZA8 [https://doi.org/10.2210/pdb6ZA8/pdb], 6ZIN [https://doi.org/10.2210/pdb6ZIN/pdb]. Source data are provided with this paper.

Research involving human participants, their data, or biological material

Policy information about studies with human participants or human data. See also policy information about sex, gender (identity/presentation), and sexual orientation and race, ethnicity and racism.

Reporting on sex and gender	N/A
Reporting on race, ethnicity, or other socially relevant groupings	N/A
Population characteristics	N/A
Recruitment	N/A
Ethics oversight	N/A

Note that full information on the approval of the study protocol must also be provided in the manuscript.

Field-specific reporting

Please select the one below that is the best fit for your research. If you are not sure, read the appropriate sections before making your selection.

☒ Life sciences ☐ Behavioural & social sciences ☐ Ecological, evolutionary & environmental sciences

For a reference copy of the document with all sections, see nature.com/documents/nr-reporting-summary-flat.pdf

Life sciences study design

All studies must disclose on these points even when the disclosure is negative.

Sample size	These are not population studies so no sample size calculation was performed. Sample size of 3 (as a minimum) was chosen for experiments to ensure reproducibility.
Data exclusions	No data excluded
Replication	Cell assays performed n=3 (Fig S3 except Fig S3a n=4).<br>NMR spectra are representative data. Data presented are from samples made from different preparations. The NMR data in Figure S5 were collected on independent samples and were not used in other experiments. The STD experiments (Figure 1a,b,d,e) were collected on fresh samples of receptor from new preparations. Each spectrum in Figure 2, S6, S8, S9 was collected on samples made from new preparations.<br>HDX MS acquired on n=4 for NT and n=3 for SR142948A per time-point.<br>3-12 time traces were collected for each ligand concentration in stopped-flow experiments.
Randomization	Not applicable. No organisms or subjects that require randomization were analysed.
Blinding	Not applicable. No organisms or subjects that require blinding were analysed.

Reporting for specific materials, systems and methods

We require information from authors about some types of materials, experimental systems and methods used in many studies. Here, indicate whether each material, system or method listed is relevant to your study. If you are not sure if a list item applies to your research, read the appropriate section before selecting a response.

Materials & experimental systems

n/a	Involved in the study	
☒	☐	Antibodies
☐	☒	Eukaryotic cell lines
☒	☐	Palaeontology and archaeology
☒	☐	Animals and other organisms
☒	☐	Clinical data
☒	☐	Dual use research of concern
☒	☐	Plants

Methods

n/a	Involved in the study	
☒	☐	ChIP-seq
☒	☐	Flow cytometry
☒	☐	MRI-based neuroimaging

Eukaryotic cell lines

Policy information about cell lines and Sex and Gender in Research

Cell line source(s)	HEKT (internal stock), HEKF- Commercial source, ordered in August 2016 from Invitrogen (Cat #: R790-07). Cells expanded, passaged and many aliquots cryopreserved in 2016. Batch used here was expanded from one of these aliquots.
Authentication	Cell lines not authenticated
Mycoplasma contamination	Cells used in this study tested negative for mycoplasma.
Commonly misidentified lines (See ICLAC register)	no commonly misidentified cell lines were used

Plants

Seed stocks	N/A
Novel plant genotypes	N/A
Authentication	N/A
